# Practical Hints for Medical Motorists

**Published:** 1909-07-10

**Authors:** 


### Motoring Notes.

### PRACTICAL HINTS FOR MEDICAL MOTORISTS.

### Construction of Inspection Pit.

An inspection pit should be of the following dimensions: Length, 6 ft.; width, 3 ft. 6 in.; and depth, about 4 ft. 6 in. A "rabbet" or ledge about 1½ in. in width should be left along the edge of the pit upon which the cover boards may rest when the pit is not in use. Steps should be provided at one end, if not at both, and efficient drainage should be provided for. A very useful addition is a piece of 1-in. batten nailed along the side of the pit throughout its entire length, at a height of about 30 inches from the bottom of the pit. The board resting and sliding on these battens forms a very handy and movable shelf, upon which one can place tools, and if made sufficiently strong it can also be used as a seat for the operator.

Where electric light is available a cable should be laid down to the pit, and one or two wall-sockets placed along the side of the pit, so that portable lamps can be connected. On no account should any other light, except a safety lamp of the Davy or miner's type, be used in the pit. Petrol vapour is supposed by many to be lighter than air, and is therefore thought to rise upward rather than accumulate in lower strata of the atmosphere. This is not the case, however, as petroleum spirit vapour is heavier than air, and always accumulates lower down. It is always well to see that the cover is put over the pit when it is not in use, lest someone, through ignorance, forgetfulness, or carelessness, should fall in.

### Economy in Petrol.

The distance which one driver can cover upon a given quantity of spirit is often a subject of much astonishment to the driver of another car of similar weight, strength, and build, who for his part has found it almost impossible to obtain corresponding results with his own vehicle, although, so far as can be seen, the two cars are otherwise identical. The whole explanation lies in the fact that the motorist who travels the greater number of miles upon a definite quantity of petrol has been taught or has discovered the correct method of running his engine. This is, in essence, the principle that the carburettor must be allowed to take in as much air as it possibly can, provided always that the maintenance of a good mixture is still secured. The most effective mixture of spirituous vapour and air is that which will drive the engine at its highest power, and this power is in no way augmented by increasing the richness of the mixture.

The real significance of the above assertion can easily be ascertained by any motor-car owner in the following manner: Close down the air opening to the carburettor, so as to obtain a rich mixture for starting the engine, and then turn the starting handle, when the motor will begin to work. Attention should now be turned to the air inlet. Open this slowly, and if a governor is fitted this should be put out of action by pulling up or pushing down the accelerator, as the case may be. As the mixture assumes its better proportion the engine will perceptibly quicken its speed, and as it gains in speed so, of course, will its power increase. Continue opening the air inlet until this is wide open, and if there is no marked diminution in the speed of the engine it may be assumed that it is now running on the least proportion of petrol obtainable. If, on the other hand, the engine begins to slow down, the air inlet should be closed down again until the engine picks up its previous speed and gives out the note that means power. The engine is now running to its best advantage, and is consuming the smallest possible amount of petrol. With such a mixture the tendency to overheat is very considerably lessened, and thereby the lubricating oil is given the best possible chance of carrying out its functions to the greatest advantage. In this way a saving is effected in the consumption both of petrol and of lubricating oil, although the latter is, of course, but a trifle compared with the former.

### Carriage of Spare Tyres.

Most drivers when starting on a long tour, and especially if their tyre covers show signs of wear, take care to provide themselves with a spare cover and tube. The chief trouble in connection with the spare covers is that no suitable place is provided for carrying them on many cars. Most owners, for want of a better place, fasten them on the back of the car, and very often carry a spare tube, partially inflated, inside the cover, under the impression that they could not have a better place than this in which to carry it, against the time when it is needed for repair or replacement. In reality no position could be worse for carrying an air tube than inside a cover, where it is, especially if fastened at the rear of the car, a prey to all the dust and dirt thrown up by the hind wheels. This dust, with its small particles of grit, is carried inside the cover and settles at the bottom, between the air tube and the cover. Here it acts like a rough file upon the surface of the air tube; for whilst the vibration of the car rubs the surfaces of the air tube and cover together, the particles of dust lying between them act like emery paper upon their fabric. Air tubes should on no account be carried in this way. They should be folded up and the valve should be well dusted with French chalk and wrapped up in rag and placed in a special bag.

Where the type of body permits, a drawer or false floor should be provided beneath the rear floor boards, wherein a spare tyre may be carried in safety. In this connection it may be mentioned that some motorists who are very careful to provide themselves with spare tubes and covers quite overlook the necessity of carrying, in addition, spare valve parts, such as rubber washers, plugs, etc., and so forth. A supply of these should never be wanting on a car.

